# 

**Published:** 1849-08

**Authors:** 


Arrata—Page 174, 3d. line from top, for "oder" read "abound."

